# Approach to urinary tract infections

**DOI:** 10.4103/0971-4065.59333

**Published:** 2009-10

**Authors:** M. S. Najar, C. L. Saldanha, K. A. Banday

**Affiliations:** Department of Nephrology, Sher-i-Kashmir Institute of Medical Sciences, Soura, Srinagar, J&K, India; 1 Department of Gynaecology and Obstetrics, Sher-i-Kashmir Institute of Medical Sciences, Soura, Srinagar, J&K, India; 2 Department of SKIMS Medical College, Bemina, Srinagar, J&K, India

**Keywords:** Acute urethral syndrome, bacteriuria, imaging studies, low-dose prophylaxis, urinary tract infection, urine culture

## Abstract

Urinary tract infection (UTI) is the most common infection experienced by humans after respiratory and gastro-intestinal infections, and also the most common cause of both community-acquired and nosocomial infections for patients admitted to hospitals. For better management and prognosis, it is mandatory to know the possible site of infection, whether the infection is uncomplicated or complicated, re-infection or relapse, or treatment failure and its pathogenesis and risk factors. Asymptomatic bacteriuria is common in certain age groups and has different connotations. It needs to be treated and completely cured in pregnant women and preschool children. Reflux nephropathy in children could result in chronic kidney disease; otherwise, urinary tract infections do not play a major role in the pathogenesis of end-stage renal disease. Symptomatic urinary tract infections occur most commonly in women of child-bearing age. Cystitis predominates, but needs to be distinguished from acute urethral syndrome that affects both sexes and has a different management plan than UTIs. The prostatitis symptoms are much more common than bacterial prostatic infections. The treatment needs to be prolonged in bacterial prostatitis and as cure rates are not very high and relapses are common, the classification of prostatitis needs to be understood. The consensus conference convened by National Institute of Health added two more groups of patients, namely, chronic prostatitis/chronic pelvic pain syndrome and asymptomatic inflammatory prostatitis, in addition to acute and chronic bacterial prostatitis. Although white blood cells in urine signify inflammation, they do not always signify UTI. Quantitative cultures of urine provide definitive evidence of UTI. Imaging studies should be done 3-6 weeks after cure of acute infection to identify abnormalities predisposing to infection or renal damage or which may affect management. Treatment of cystitis in women should be a three-day course and if symptoms are prolonged, then a seven day course of antibiotics should be given. Selected group of patients benefits from low-dose prophylactic therapy. Upper urinary tract infection may need in-patient treatment. Treatment of acute prostatitis is 30-day therapy of appropriate antibiotics and for chronic bacterial prostatitis a low dose therapy for 6-12 months may be required. It should be noted that no attempt should be made to eradicate infection unless foreign bodies such as stones and catheters are removed and correctable urological abnormalities are taken care of. Treatment under such circumstances can result only in the emergence of resistant organisms and complicate therapy further.

## Introduction

Urinary tract infection (UTI) is the third most common infection experienced by humans after respiratory and gastro-intestinal infections. In fact, bacterial infections of the urinary tract are the most common cause of both community acquired and nosocomial infections for patients admitted to hospitals in United States. It is distressing and occasionally life threatening. However, the prognosis and management of urinary tract infections depends on the site of infection and any predisposing factors.

UTI may be defined as a condition in which bacteria are established and multiplying within the urinary tract. Diagnosis requires demonstration of bacteriuria. Exceptions to this include patients with pyogenic abscess of kidney or perinephric tissue, obstructed pyonephrosis or bacterial prostatitis in whom the urine may be sterile.

Some definitions are necessary because the infection of the urinary tract may result from microbial invasion of any of the tissues extending from urethral orifice to the renal cortex. Although the infection and resultant symptoms may be localized, the presence of bacteria in urine places the entire urinary system at risk of invasion by bacteria.

## Significant bacteriuria

It is defined as the presence of 100 000 or more colony forming units (CFU) per ml of urine. This Kass[1] criteria has been questioned and bacterial counts of 10^2^ or more organism per ml particularly when accompanied by pyuria (>10 wbc/mm^3^) provide impressive evidence of urinary tract infection in symptomatic young women.[2] The Infectious Disease Society of America (IDSA) gave a slightly more relaxed consensus definition requiring 10^3^ organisms per ml to diagnose cystitis and 10^4^ per ml for pyelonephritis.[3]

## Anatomic location

It is useful to distinguish between upper (kidney) and lower (bladder, prostate and urethra) urinary tract infections. Infections confined to lower urinary tract commonly cause dysuria, frequency and urgency. Pyelonephritis (inflammation of the renal parenchyma) is a clinical syndrome characterized by chills and fever, flank pain and constitutional symptoms caused by bacterial invasion of the kidney.

The localization of the site of infection on the basis of symptoms and signs can be inaccurate. Using ureteral catheterization, it has been shown that approximately 50% of women with asymptomatic bacteriuria had infection in their upper tracts.[4]

Response to treatment is now used to distinguish between the two upper versus lower urinary tract infections. This is based on the observation that many women with symptoms of cystitis shown by localization studies to be confined to bladder can be cured by a single dose of antibiotic.[5] Recurrence of bacteriuria with the same organism within seven days of single dose therapy was reported to be most often associated with upper tract infection.

## Complicated and uncomplicated urinary tract infection

**Figure 1:**
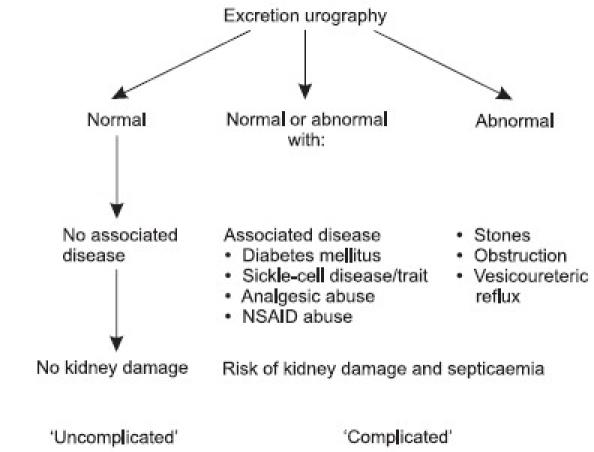
Classification of complicated and uncomplicated urinary tract infection. (Adapted from reference 6)

There is a general agreement that for the best management of patients with urinary tract infections, it is important to distinguish between complicated and uncomplicated infections. Complicated infections include those involving the parenchyma (pyelonephritis or prostatitis) and frequently occur in the setting of obstructive uropathy or after instrumentation. The presence of obstruction, stones or high-pressure vesico-ureteric reflux, perinephric abscess, life-threatening septicemia or a combination of these predispose to kidney damage [Figure 1].[6] Episodes may be refractory to therapy, often resulting in relapses and occasionally leading to significant sequelae such as sepsis, metastatic abscess and rarely acute renal failure. An uncomplicated infection is an episode of cysto-urethritis following bacterial colonization of the ureteral and bladder mucosae. This type of infection is considered to be uncomplicated because sequelae are rare and exclusive due to the morbidity associated with reinfection in a subset of women. A subset of patients with pyelonephritis (acute uncomplicated pyelonephritis), namely, young women who respond well to therapy may also have a low incidence of sequelae.

## Recurrent infection-reinfection, relapse and treatment failure

Reinfection is a recurring infection due to a different microorganism that is usually drug susceptible. Most recurring episodes of cysto-uretheritis are due to reinfections that are much more common than relapse and accounts for about 80% of recurrent infections.[7] Unlike relapse, reinfection does not represent failure to eradicate infection from urinary tract but is due to reinvasion of the system. Prophylactic measures must be initiated. Relapse is a return of infection due to the same micro-organism which is often drug resistant. It is defined as the recurrence of bacteriuria with the same organism within three weeks of completing treatment, which during treatment rendered the urine sterile. Relapse implies that there has been a failure to eradicate the infection. This most often occurs in association with renal scars, stones, cystic disease or prostatitis and in patients with chronic interstitial disease or in those who are immune compromised.[8]

The term treatment failure has been used to describe failure to eradicate bacteriuria during treatment and failure to prevent relapse.

Factors predisposing to treatment failure:

- Recent antibiotic treatment
- Hospital acquired infection
- Renal or bladder calculi
- Obstructive uropathy
- Renal cysts
- Renal diseases such as reflux nephropathy, chronic interstitial nephropathy, analgesic nephropathy, diabetic nephropathy, sickle cell nephropathy, immunosuppression, and prostatitis.

## Risk factors and pathogenesis

The understanding of the pathogenesis and epidemiology of urinary tract infections can facilitate early recognition and possible prevention.

Associations have been established between UTI and age, pregnancy, sexual intercourse, use of diaphragm and a spermicide, delayed post-coital micturition, menopause and a history of recent UTI. Factors that do not seem to increase the risk of UTI include diet, use of tampons, clothing and personal hygiene including methods of wiping after defecation and bathing practices.[9]

Studies on pathogenesis have elucidated specific interactions between the host and microbes that are causally related to bacteriuria. Bacteria in the enteric flora periodically gain access to the genitorurinary tract. Close proximity of anus in women to peri-urethra is a likely factor. Bacterial colonization of periurethra often precedes the onset of bladder bacteriuria. P-fimbriated strains of *Escherichia coli* adhere to uroepithelial cells in which glycolipids function as receptors in women who secrete blood group antigens. Opposing colonization are several host factors, most notably acid pH, normal vaginal flora and type specific cervico-vaginal antibodies.[7]

After periurethral colonization, uropathogens gain access to the bladder via the urethra, to kidney via ureters and to prostate via the ejaculatory ducts. The urethra and uretero-vesicle junction are mechanical barriers that prevent ascension. In the bladder, the organisms multiply, colonize the bladder mucosa and invade mucosal surface. Although urine adequately supports the growth of most uropathogens, the bladder has several mechanisms that prevent bacteriuria.

1. A mucopolysaccaride (urine slime) layer covers the bladder epithelium and prevents elonization.
2. Tamm-Horsfall protein which is a component of uromucoid adheres to P-fimbria and prevents colonization.
3. Urine flow and bladder contraction serve to prevent stasis and colonization.

Bladder infection sets the stage for subsequent migration to the kidneys where organisms such as P-fimbriated *E. coli* adhere to renal tubular cells. Outside the setting of obstructive uropathy, this strain of *E. coli* is the most common cause of pyelonephritis. With obstruction, bacterial adherence is unimportant. Other host factors that prevent a renal infection are high osmolality, high ammonium concentration, phagocytes and high urine flow rate.[10]

## Clinical setting

### Asymptomatic bacteriuria

This is especially common in women as evidenced by a minimum prevalence of 2-4% in young and 10% in elderly women. The cumulative prevalence of asymptomatic bacteriuria in women increases about 1% per decade throughout life regardless of ethnicity and geographic locations.

In contrast to women, the occurrence of asymptomatic bacteriuria in men is rare until after 55 years of age, at which time the prevalence increases per decade and approaches the rate in elderly women. Prostatic hypertrophy and increased likelihood of instrumentation account for the bacteriuria in older men.[11]

Differences between men and women in the rates of bacteriuria have been attributed to the shorter female urethra and its proximity to the vagina and rectal mucosa and their abundant microbial flora.

### Symptomatic urinary tract infection

These occur in all age groups. Among newborns and infants, boys are affected more than the girls. When urinary tract is the source of neonatal sepsis, serious underlying congenital anomalies are frequently present.

**Figure 2:**
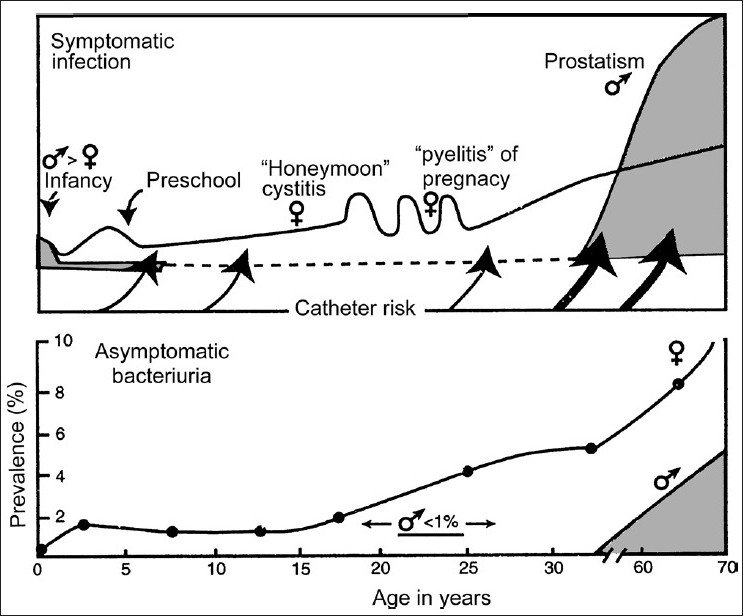
Frequency distribution of symptomatic urinary tract infection and prevalence of asymptomatic bacteriuria by age and sex (Male – shaded area; Female – line)

During childhood, persistent bacteriuria with or without repeated symptomatic episodes occurs in a small group (less than 2%) of school-aged girls. Such girls and also school-aged boys with bacteriuria should have a urological evaluation to detect correctable structural abnormalities when UTIs are documented [Figure 2].[11]

Sexually active women have a markedly increased risk of cystitis. Vast majority of acute symptomatic infections involve young women. A prospective study demonstrated an annual incidence of 0.5-0.7 episodes per patient year in this group.[12] In the absence of prostatitis, bacteriuria and symptomatic UTIs are unusual in men. The risk of cystitis in young men due to uropathogenic *coli* increases because of lack of circumcision or having a partner with vaginal colonization with such P-fimbriated *E. coli*. At any age, both sexes may develop symptomatic infections in the presence of risk factors that alter urinary flow. These include:[13]

1. Congenital anomalies
2. Renal calculi
3. Ureteral occlusion (partial or total)
4. Vesico-ureteral reflux
5. Residual Urine in bladder
  Neurogenic bladder
  Urethral stricture
  Prostatic hypertrophy
6. Instrumentation of urinary tract
  Indwelling urinary catheters
  Catheterization
  Urethral dilatation
  Cystocopy

## Clinical Features

### Acute urethral syndrome

The cardinal symptoms of frequency and dysuria occur in more than 90% of ambulatory patients with acute genitourinary tract infections. However, one-third to one- half of all these patients do not have significant bacteriuria, although most have pyuria. These patients have acute urethral syndrome which can mimic both bladder and renal infections. Vaginitis, urethritis and prostatitis are common causes of the acute urethral syndrome.[14]

### Vaginitis

The presence of an abnormal vaginal discharge (leucorrhoea) and irritation makes vaginitis the likely cause of dysuria unless a concomitant UTI can be confirmed by culture. Candida albicans, the most common specific cause of vaginitis, can be demonstrated by culture or by finding yeast cells in a gram-stained smear of vaginal secretions or in a saline preparation with the addition of potassium hydroxide.

Trichomoniasis can be documented with a saline preparation that shows the motile protozoa of trichomonas vaginitis. Generally, nonspecific vaginitis is associated with gardenerella vaginitis. A clue of this diagnosis is the presence of many small Gram-negative bacilli that adhere to vaginal epithelial cells.

### Urethritis

Acute urinary frequency, dysuria and pyuria in the absence of vaginal symptoms favor the diagnosis of urethritis or UTI. *Chlamydia trachomatis* is the common cause of the acute urethral syndrome in women and of nonspecific urethritis in men. Neisseria gonorrhoeae is an important cause of urethritis and dysuria. Herpes simplex virus, usually type 2, is another sexually transmitted agent that can cause severe dysuria through ulceration in close proximity to the urethral orifice. The diagnosis of Herpes progenitalis can be confirmed by finding giant multinucleated transformed cells in epidermal scrapings stained with Wright's stain (Tzanck Smear), by isolating the virus in tissue cultures or by direct fluorescent antibody test.

### Prostatitis

Prostatitis is a common problem in men that causes dysuria and urinary frequency in middle-aged and younger men more frequently than urinary tract infection do. Prostate syndromes have classically been divided into four clinical entities

- Acute bacterial prostatitis
- Chronic bacterial prostatitis
- Nonbacterial prostatitis
- Prostatodynia

Recently, consensus classification of prostatitis syndromes has come up. This classification includes four categories and two subcategories.[15]

- Acute bacterial prostatitis;
- Chronic bacterial prostatitis;
- Chronic prostatitis/chronic pelvic pain syndrome (CP/CPPS);
- Asymptomatic inflammatory prostatitis.
- CP/CPPS has been divided in to two sub-categories:
  1. Inflammatory CP/CPPS; and
  2. Non- inflammatory CP/CPPS

Acute bacterial prostatitis: The patient often appears acutely ill with the sudden onset of chills and fever, urinary frequency and urgency, dysuria, perineal and low back pain and constitutional symptoms. Rectal examination should be avoided because of the risk of precipitating sepsis, but may disclose a tender, hot and swollen prostate. Microscopic examination of the urine usually displays numerous white cells. Urine culture is usually positive for enteric Gram-negative bacteria and Gram-positive bacteria staphylococci and enterococci are less frequently isolated.

Chronic bacterial prostatitis: Relapsing UTIs is a hallmark of chronic bacterial prostatitis. Urinary frequency, dysuria, nocturia and low back and perineal pain are the usual symptoms, although patients may have a minimum of symptoms between UTIs. The patient is often afebrile, does not appear acutely ill, and may have an unremarkable prostate examination. Initially, there is a negative midstream urine examination and culture but after prostate massage, the urine is positive for white blood cells and culture grows a uropathogen.

Nonbacterial prostatitis: This is the most common form of chronic prostatitis. It mimics chronic bacterial prostatitis clinically and displays inflammatory cells on post-prostate massage specimens. However, a bacteriological culture of urine and prostatic secretions are sterile. The etiology is unknown, but some evidence exists for an infectious cause involving organisms that are difficult to culture.

Prostatodynia: This has also been referred to as chronic noninflammatory prostatitis. Clinically, it presents with symptoms similar to other forms of chronic prostatitis. It is distinguished by the absence of inflammatory cells or uropathogens from all specimens.

Chronic prostatitis/chronic pelvic pain syndrome: The traditional classification suggested that the prostate was the cause for some patients (nonbacterial prostatitis), whereas other problems were responsible in others (prostatodynia). The characteristic symptoms for either group were very poorly defined. CP/ CPPS acknowledges the central role of pain complaints in the syndrome. Also there is inherent recognition that the prostate gland may not be responsible for every patient's symptoms. Its two subcategories are as follows:

- Inflammatory CP/CPPS: The consensus classification considers symptomatic patients without bacteriuria but who have inflammation in their expressed prostate secretions, their voided bladder 3 (VB3) or their semen fluid analysis (SFA), to have inflammatory CP/CPPS.
- Noninflammatory CP/CPPS: Patients without inflammation in their expressed prostate secretions, their voided bladder 3 (VB3) or their semen fluid analysis (SFA) are considered to have noninflammatory CP/CPPS.

Asymptomatic inflammatory prostatitis: The consensus classification also includes a category for patients with objective evidence of prostatic inflammation noted during histological evaluation of prostatic tissue. This diagnosis commonly occurs in patients who have inflammation documented during evaluation of other urologic conditions, for example, prostatic evaluated for a raised prostate-specific antigen. Another example is seminal fluid inflammation noted during evaluation from an infertile couple. The long-term consequences of such asymptomatic inflammation are unknown. Further, only limited data are available on the relative merits of antimicrobial or other therapies for such asymptomatic patients.

Urinary tract infection: Despite the mimicking syndromes, a presumptive diagnosis of infections of urinary tract can be established economically by analyzing urine in patients with characteristic signs and symptoms. Acute uncomplicated UTIs mainly occur in women of child-bearing age. The presenting features are only suggestive of the site of infection. Patients with bacterial cystourethritis, as distinct from urethritis caused by sexually transmitted disease (STD) pathogens, will have prior episodes and experienced symptoms for less than one week and will experience suprapubic pain.

## Diagnosis

### Microscopic examination of urine

In a centrifuged sediment, patients with significant bacteriuria almost always show bacilli in the urine, whereas only approximately 10% of patients with less than 10^5^ CFU per ml show bacteria. About 60-85% of patients with significant bacteriuria have 10 or more white blood cells per high power field in the segment of mid-stream urine. Also 25% of patients with negative urine cultures also have pyuria, 10 or more white blood cells per high power field and only approximately 40% of patients with pyuria have 10^5^ or more bacteria per ml of urine by qualitative cultures.

### Pyuria

95% of patients with pyuria have a genitourinary tract infection; however, pyuria cannot distinguish a bacterial UTI from acute urethral syndrome. Tuberculosis,[16] analgesic nephropathy, interstitial nephritis, perinephric abscess, renal cortical abscess, disseminated fungal infection and appendicitis may also result in pyuria.

### Gram strain

A simple Gram-stained smear can enhance the specificity of the test because morphology and stain characteristics aid in identifying the likely pathogen and in targeting empiric therapy.

### Urine culture

The diagnosis of UTI from simple cystitis to complicated pyelonephritis with sepsis can be established with absolute certainty only by cultures of urine. The major indications for urine cultures are:

- Patients with symptoms or signs of UTIs;
- Follow-up of recently treated UTI;
- Removal of indwelling urinary catheter;
- Screening for asymptomatic bacteriuria during pregnancy; and
- Patients with obstructive uropathy and stasis, before instrumentation.

Urine specimens must be cultured promptly within 2h or can be preserved by refrigeration or a suitable chemical additive (boric acid sodium formate). Acceptable methods of collection are:

- Midstream urine after careful washing;
- Urine obtained by single catheterization;
- Urine obtained by supra pubic needle aspiration; and
- Sterile needle aspiration of urine from the tube of a closed catheter drainage system.

Results of cultures depend on the clinical setting in which bacteriuria occurs. For example, *E. coli* are found in the urine of 80-90% of patients with acute uncomplicated cystitis and acute uncomplicated pyelonephritis. Many patients with staghorn calculi harbour urea-splitting proteus organisms in their urine. *Klebsiella*, *Pseudomonas* and *Enterobacter* infections are commonly acquired in the hospital. The presence of *Staphylococcus aureus* often is a clue to concomitant *Staphylococcal* bacteremia, unless an underlying risk factor exists.

Micro-organisms in young men are similar to the organisms that cause uncomplicated infections in women. Enterococci and coagulase-negative staphylococci are more common in elderly men; most likely representing recent instrumentation or catheterization. *C. albicans* is rarely encountered except in patients with indwelling catheters, nosocomial UTIs or relapsing infections after multiple courses of antibiotics. Although the likely organism and usual susceptible patterns are sufficient to guide initial empiric therapy of uncomplicated UTI, adequate treatment of acute bacterial pyelonephritis and complicated UTIs necessitates precise therapy based on isolation of the causative bacterium and its antimicrobial susceptibility.[13]

### Imaging studies

In general, imaging should be done 3-6 weeks after cure of acute infection to identify abnormalities predisposing to infection or renal damage or which may affect management.[17] Rarely, imaging is carried out in the acute phase, particularly where there is severe loin pain, to identify possible sepsis (pyonephrosis or abscess) or to differentiate acute pyelonephritis from ureteric colic. It is important to recognize that abnormalities will be found in less than 5% of unselected cases.

#### Plain X-ray of abdomen

These are used to show the presence and extent of calcification in the urinary tract. They are less sensitive in the detection of ureteric calculi. Plain films are of value in monitoring change in position, size and number of calculi.

#### Ultrasound

Ultrasound (USG) combined with plain X-ray has become the imaging method of choice in patients with recurrent infections. It is a sensitive detector of pelvicalyceal dilatation, indicative of possible obstruction. Echoes within a dilated pelvicalyceal system, either diffuse or layered, suggest the presence of pyonephrosis. Drainage of an obstructed kidney can be guided by ultrasonography. It provides accurate renal length measurements and identifies the majority of renal scars, abscesses and perinephric fluid collections.[18]

Ultrasound may show short segments of dilated ureter adjacent to the renal pelvis, at pelvic brim level or behind the full bladder. It can also assess the bladder for wall thickness, calculi, diverticula and emptying as well as assess prostate size.

#### Intravenous urography

Intravenous urography (IVU) provides anatomical detail of the calyces, pelvis and ureter not obtained from ultrasonography. Calyceal detail is essential to diagnose papillary necrosis and medullary sponge kidney and careful assessment of the calyces and overlying parenchyma is necessary to diagnose reflex nephropathy.

Gram-negative bacilli have the ability to impede ureteral peristalsis and transient abnormalities of the IVU are common with acute pyelonephritis. These include hydroureter, vesico-ureteric reflux, diminished pyelogram, loss of renal outline and renal enlargement. IVU should also be avoided for the first 6-12 weeks after pregnancy to allow resolution of the physiological dilatation of the pelvicalyceal system and ureter.

#### Computed tomography

CT is the most common method of detecting renal and ureteric calculi, including calculi that are lucent on plain radiographs. It is a sensitive detector of pelvicalyceal dilatations, renal abscesses and perinephric collections than US. Contrast enhanced CT is very sensitive for acute pyelonephritis.[19]

However, CT involves more radiation than even IVU, the potential risks of contrast media and is more expensive and less readily available than US. Therefore, it should be reserved as a second-line investigation for patients with severe infection not responding to appropriate treatment or for diagnostic problems not resolved by IVU or US.

#### Static renal scintigraphy

Di-mercapto-succinic acid (DMSA) scintigraphy is a sensitive detector of renal parenchymal infection in children.

### Indications and choice of renal imaging

#### Acute infection

Patients who have severe loin pain or whose infection does not settle on treatment should have US and plain X-ray to exclude pyonephrosis, intrarenal or perinephric sepsis or calculi. CT may be undertaken if no abnormality is seen on US in such patients. If ureteric colic is suspected, IVU or spiral CT should be used.[20]

### Imaging after treatment of infection

In women, there is no indication for imaging following a single or infrequent infection. Recurrent attacks more often than 2 per 6 months should be investigated by USG and plain KUB. In men, UTI is much less common than in women, and imaging is indicated after the first documented bacteriuria to exclude predisposing factors especially impaired bladder emptying. USG and plain film are the best first choice.[21]

Imaging should be considered if urinary infection is slow to resolve, if there is relapse or if there are risk factors for papillary necrosis. IVU is the method of choice to check for papillary necrosis, medullary sponge kidney or reflux nephropathy. IVU is also indicated in all patients over the age of 40 who have gross hematuria because of the risk of associated cancer.

#### Micturating cystourography

MCU is not usually indicated in adults with urinary infection unless they have loin or abdominal pain during voiding, suggestive of reflux or as part of the investigation of impaired bladder emptying.

### Urodynamic studies

These may be necessary in patients with unexplained impairment of bladder emptying.

## Treatment of urinary tract infection

For effective management of UTI, the following principles must be recognized.

1. Asymptomatic patients should have colony counts greater than or equal to 10^5^ per ml on at least 2 occasions before treatment is considered.
2. Unless symptoms are present, no attempt should be made to eradicate bacteriuria until catheters, stones or obstructions are removed.
3. Selected patients with chronic bacteriuria may benefit from suppressive therapy.
4. A patient who develops bacteriuria as a result of catheterization should be treated to re-establish sterile urine.
5. Efficacy of treatment should be evaluated by urine culture, one week after completion of therapy except in nonpregnant adult women with uncomplicated cystitis and uncomplicated pyelonephritis who respond to therapy.

### Asymptomatic bacteriuria

#### Pregnancy

Pregnancy increases the risk of UTI complications. The rate of prematurity in children born to women who have bacteriuria during pregnancy is increased, and 20-40% of these patients develop pyelonephritis. Successful therapy of these patients with bacteriuria decreases the risk of symptomatic infection by 80-90%. Therefore, all women should be screened twice during pregnancy for asymptomatic bacteriuria. All bacteriuric patients should be treated for seven days, with follow-up cultures to identify relapses. In selecting therapy, risk to foetus should be considered. Amoxicillin or cephalexin usually suffice.[22]

#### Children

Asymptomatic bacteriuria in young children and school-aged girls may signify underlying vesicoureteral reflux. Therefore, asymptomatic bacteriuria should be treated with follow-up urologic evaluation after six weeks.

#### General population

Asymptomatic bacteriuria in men and nonpregnant women, a common condition in the elderly,[23] does not appear to cause renal damage in the absence of obstructive uropathy or vesicoureteral reflux and therefore it should not be treated.

Instrumentation of genitourinary tract should be avoided in patients with asymptomatic bacteriuria or, if necessary done under the cover of prophylactic antibiotic therapy. Selected high-risk patients (renal transplantation or neutropenia) may benefit from therapy for asymptomatic bacteriuria.

#### Diabetis mellitus

Patients with asymptomatic bacteriuria who have conditions predisposing to papillary necrosis such as diabetis mellitus must be considered at risk of potentially harmful extension of infection to the kidney which may accelerate interstitial damage. Treatment is similar to that used for sysmptomatic patients.

### Uncomplicated cystitis

This is almost exclusively a disease of sexually active women mostly between the ages of 15 and 45 years. Although reinfection is common, complications are rare.

#### Short course therapy

Infections truly confined to bladder or urethra respond as well to single-dose or short-course (3 day) therapy as to conventional therapy for 10-14 days. However, it has been observed that three- day therapy is more effective than single-dose therapy.[24] A three-day regimen of amoxillin-clavulinate was found to be significantly less effective than a three-day regimen of ciprofloxacin in treating uncomplicated UTIs in women.[25] However, resistance has increased to various antimicrobials and more than one quarter of *E. coli* strains causing acute cystitis are resistant to amoxicillin, sulfa drugs and cephalexin and resistance to co-trimoxazole is now approaching these levels. Resistance to fluoroquinolones is also rising. Thus, knowledge of local resistance pattern is needed to guide empirical therapy.[26]

#### Seven-day regimen

A longer course of therapy for cystitis should be given to patients with complicating factors that lead to lower success rates and a higher risk of relapse. These factors include a history of prolonged symptoms (more than seven days), recent UTI, diabetes, age above 65 years and use of a diaphragm. Importantly, both elderly and diabetic women frequently have concurrent renal infection, thus short course therapy should not be used in them.

### Recurrent cystitis (re-infections)

Some women especially whose periurethral and vaginal epithelial cells avidly support attachment of coli-form bacteria suffer from recurrent episodes of cystitis in the absence of recognized structural abnormalities of the urinary tract. Management in such women include the following:

1. Post-coital prophylaxis
2. Continuous low dose prophylaxis and
3. Self-administered therapy.

Postcoital prophylaxis is the most helpful for patients who associate recurrent UTIs with sexual intercourse. In these women, a single dose of an antimicrobial after sexual intercourse significantly reduces the frequency of UTIs.

Women with recurrent UTIs (more than three UTIs per year) benefit from thrice weekly bed time antibiotic therapy. Such therapy significantly reduces the frequency of episodes of cystitis from an average of 3 per patient-year to 0.1 per patient-year.[27] This regimen is known as continues low dose prophylaxis.

Women with fewer than three UTIs per year can be offered self-administered treatment. At the first sign/symptom of a UTI, such women should take a single-dose regimen of TMP-SMX or a fluoroquinolone. This is both effective and well tolerated.[28]

Several prospective studies have demonstrated the efficacy of either nitrofurantoin 50 mg or nitrofurantoin macrocrystals 100 mg at bed time for prophylaxis against recurrent reinfection of urinary tract. Such a regimen has little if any effect on the faecal flora and presumably acts by providing intermittent urinary antibacterial activity.

Perhaps, the most popular prophylactic regimen currently used in women susceptible to recurrent UTI is low-dose TMP-SMX; as little as half a tablet (trimethoprim, 40 mg, sulfamethoxazole, 200 mg) three times weekly at bed-time is associated with an infection frequency of less than 0.2 per patient-year. The efficacy of this prophylactic regimen appears to remain unimpaired even after several years. Similar to TMP-SMX, the fluoroquinolones may be used in a low-dose prophylactic regimen. The efficacy of these regimens is further delineated by their potency in preventing UTI in the far challenging population of kidney transplant recipients.

### Acute bacterial pyelonephritis

In this setting, blood and urine cultures should be obtained.

#### Out-patient therapy

For uncomplicated acute pyelonephritis, a fluoroquinolone or co-trimoxazole is the drug of choice for initial therapy. After culture results are available, a full 10-14 day course of the antimicrobial to which the organism is susceptible should be instituted.[29]

#### In-patient therapy

Patients who require admission to the hospital should be treated initially with a third-generation cephalosporin or a fluoroquinolone and gentamicin 4-7 mgs every 24 h if the urine shows Gram-negative bacilli on microscopy. If gram-positive cocci are seen in the urine, intra-venous ampicillin 1g every 4 hours should be given in addition to gentamicin, to cover the possibility of enterococcal infection. If no complications ensue and patient becomes afebrile, the remaining two-week course can be completed with oral therapy.

However, persistent fever, persistent bacteriuria in 48-72 h or continual signs of toxicity beyond three days of therapy suggest the need for evaluation to exclude obstruction, metastatic focus or formation of a perinephric abscess. Adequate fluids must be given to maintain adequate arterial perfusion. Failure to respond to seemingly appropriate therapy suggests the possibility of underlying pus. Examination by US or CT may disclose an obstructed ureter or perinephric abscess, both of which require surgical drainage.[30]

### Recurrent renal infections (Relapses)

Chronic bacterial pyelonephritis is one of the most refractory problems as relapse rates are as high as 90% occur.

#### Risk factors

To improve the success rate, it is important to repair any correctable lesions, that obstructions to urine flow be relieved and that foreign bodies (indwelling urinary catheters or renal staghorn calculi) be removed.

If the risk factors cannot be corrected, long-term eradication of bacteriuria is almost impossible. To attempt eradication in such instances leads only to the emergence of more resistant strains of bacteria or fungi. In such case, one must be resigned to treatment of symptomatic episodes of infection and to suppression of bacteriuria in selected patients.

#### Acute symptomatic infection

The treatment of acute symptoms and signs of UTI in a patient with chronic renal bacteriuria is the same as for patients with acute bacterial pyelonephritis.

#### Prolonged treatment

Some patients with relapsing bacteriuria respond to six weeks of antimicrobial therapy. This is especially true of patients with no underlying structural abnormality and of men with normal prostatic examination.

#### Suppressive therapy

Patients who fail the longer therapy, who have repeated episodes of symptomatic infection or who have progressive renal disease despite corrective measures, are candidates for suppressive antibiotic therapy. These patients should have two to three days of specific high-dose antimicrobial therapy to which their infecting bacteria are susceptible to reduce the colony counts in their urine. The preferred agent for long-term suppression is methenamine mandelate. Alternative therapy is cotrimoxazole, two tablets twice daily or nitrofurantion 50-100 mg twice daily.[31]

#### Prognosis

Although UTIs are a common cause of appreciable morbidity, they do not play a major role in the pathogenesis of end-stage renal disease. Patients who come to renal replacement therapy because of chronic bacterial pyelonephritis almost always have an underlying structural defect. Most often, the lesion is chronic atrophic pyelonephritis associated with vesicoureteric reflux that started in infancy. The role of surgical correction of vesicoureteral reflux is not clear, but what is certain, is the importance of meticulous control of infection in children to prevent progressive renal scarring and renal failure by early adulthood.

### Prostatitis

#### Acute bacterial prostatitis

The drug of choice is cotrimoxazole or fluoroquinolone. However, treatment must be ultimately based on an accurate microbiological diagnosis and continued for 30 days to prevent chronic bacterial prostatitis. Urethral catheterization should be avoided. If acute urinary retention develops, drainage should be by supra-public needle aspiration or if prolonged bladder drainage is required by a suprapubic cystostomy tube.

#### Chronic bacterial prostatitis

The hallmark of chronic bacterial prostatitis is relapsing UTI. It is most refractory to treatment. Although erythromycin with alkalinization of urine is effective against susceptible Gram-positive pathogens, most instances of chronic bacterial prostatitis are caused by gram-negative enteric bacilli. Cotrimoxazole or fluoroquinolone is the drug of choice.

Approximately 75% of patients improve and 33% are cured with 12 weeks of cotrimoxazole therapy. For patients who cannot tolerate cotrimoxazole or fluoroquinolone, nitrofurantoin 50 or 100 mg once or twice daily can be used for long-term (6-12 months) suppressive therapy.[32]

#### Nonbacterial chronic prostatitis

Therapy is difficult because an exact etiology has not been identified. Owing to a concern for *C. trachomatis, Ureaplasma urealyticum* and other fastidious and difficult to culture organism, many experts recommended a six- week trial of tetracycline or erythromycin. Symptomatic therapy with NSAIDs and alpha-receptor blockers has also been used.

#### Catheter-associated infection

Urinary catheters are valuable devices for enabling drainage of the urinary bladder but their use is associated with an appreciable risk of infection. For a single (in-and-out) catheterization, the risk is small (12%), though this prevalence is much higher in diabetic and elderly women. However, bacteriuria occurs in virtually all patients with indwelling catheters within three to four days unless placement is done under sterile conditions and a sterile, closed drainage system is maintained. The use of a neomycin-polymyxin irrigate does not prevent catheter-associated infection.

Catheter-associated bacteriuria should only be treated in the symptomatic patient. When the decision to treat is made, removal of the catheter is an important aspect of therapy, because if an infected catheter remains in place, relapsing infection is very common. The interaction between the organisms and catheter cause the organism to form a biofilm, an area in which antibiotics are unable to completely eradicate these organisms. The empiric therapy of these infections is similar to that of complicated UTIs. Patients who rapidly respond to the therapy may be treated only for seven days.

The use of catheters impregnated with antimicrobial agents reduces the incidence of asymptomatic bacteriuria in patients catheterized for less than two weeks. Despite precautions, the majority of patients catheterized for more than two weeks eventually develop bacteriuria.[33]

### Fungal urinary tract infection

The most common form of fungal infection of urinary tract is that caused by Candida species. Such infections usually occur in patients with indwelling catheters who have been receiving broad-spectrum antibiotics, particularly if diabetes mellitus is also present or corticosteroids are being administered. Although most of these infections remain limited to the bladder and clear with the removal of the catheter, cessation of antibiotics and control of diabetes mellitus, the urinary tract is the source of approximately 10% of episodes of candidemia, usually in association with urinary tract manipulation or obstruction.[34] Spontaneously occurring lower UTI caused by Candida species is far less common, although papillary necrosis, caliceal invasion and fungal ball obstruction have all been described as resulting from ascending candidal UTI that is not related to catheterization.

Hematogenous spread to the kidney and other sites within the genitourinary tract may be seen in any systemic fungal infection, but it occurs particularly in coccidioidomycosis and blastomycosis.[35] In immunosuppressed patients, a common hallmark of disseminated cryptococcal infection is the appearance of this organism in the urine. *Cryptococcus neoformans* commonly seeds the prostate and far less commonly may cause a syndrome of papillary necrosis, pyelonephritis and pyuria akin to that seen in tuberculosis.

There are no criteria to distinguish between colonization or infection with candiduria, so the following approach is adopted for the treatment.

In patients with catheter-associated candidal UTI, removal of the preceding catheter, insertion of a three-way catheter and infusion of an amphotericin rinse for a period of three to five days eradicates greater than 50% infections.[36] In patients with candiduria without an indewelling catheter, fluconazole 200 to 400 mg /day for 10 to 14 days should be given. In a population of organ transplant patients, such an approach has been successful in more than 75% of patients with candiduria.[37]

Any patient with candiduria who has to undergo instrumentation of the urinary tract requires systemic therapy with amphotericin or fluconazole to prevent the consequences of transient candidemia.
